# End-tidal CO_2_ Monitoring is Available in Most Community Hospitals in a Rural State: A Health System Survey

**DOI:** 10.5811/westjem.2018.12.40554

**Published:** 2019-02-14

**Authors:** Steven A. Ilko, J. Priyanka Vakkalanka, Azeemuddin Ahmed, Daniel A. Evans, Hans R. House, Nicholas M. Mohr

**Affiliations:** * University of Iowa Carver College of Medicine, Department of Emergency Medicine, Iowa City, Iowa; † University of Iowa College of Public Health, Department of Epidemiology, Iowa City, Iowa; ‡ University of Iowa Tippie College of Business, Iowa City, Iowa; § Indiana University, Department of Emergency Medicine, Indianapolis, Indiana; ¶ University of Iowa Carver College of Medicine, Division of Critical Care, Department of Anesthesia, Iowa City, Iowa

## Abstract

**Introduction:**

Procedural sedation and analgesia (PSA) provides safe and effective relief for pain, anxiety and discomfort during procedures performed in the emergency department (ED). Our objective was to identify hospital-level factors associated with routine PSA capnography use in the ED.

**Methods:**

This study was a cross-sectional telephone survey of ED nurse managers and designees in a Midwestern state. Respondents identified information about hospital infrastructure, physician staffing, family practice (FP) physicians only, board-certified emergency physicians (EPs) only (or both), and critical intervention capabilities. Additional characteristics including ED volume and hospital designation (i.e., rural-urban classification) were obtained from the Centers for Medicare and Medicaid Services and the state hospital association database, respectively. The primary outcome was reported use of PSA capnography. We conducted univariate analyses (relative risks, 95% confidence interval [CI]) to identify associations between hospital-level characteristics and PSA capnography use.

**Results:**

We had an overall response rate of 98% (n=118 participating hospitals). The majority of EDs were in rural settings (78%), with a median of 5,057 visits per year (interquartile range 2,823–14,322). Nearly half of the EDs were staffed by FP physicians only, while 16% had board-certified EPs only. Nearly all hospitals (n=114, 97%), reported using continuous capnography for ventilated patients, and 74% reported use of capnography during PSA. Urban hospitals were more likely to use PSA capnography than critical access hospitals (relative risk 1.45; 95% CI, 1.22–1.73), and PSA capnography use increased with each ED volume quartile. Facilities with only EPs were 1.46 (95% CI, 1.15–1.87) times more likely to use PSA capnography than facilities with FP physicians only.

**Conclusion:**

Continuous capnography was available in nearly all EDs, independent of size, location or patient volume. The implementation of capnography during PSA was less penetrant. Smaller, rural departments were less likely than their larger, urban counterparts to implement these national guidelines. Rurality and hospital size may be potential institutional barriers to capnography implementation.

*Throughout this paper, the authors use “BCEM” as an acronym/abbreviation for “Board-Certified in Emergency Medicine.” This includes certification by the American Board of Emergency Medicine and the American Osteopathic Board of Emergency Medicine, both of which require completion of residency training in emergency medicine. This acronym does not refer to the American Board of Physician Specialties (ABPS) designation of “Board Certification in Emergency Medicine,” which can be attained without residency training in emergency medicine*.

## INTRODUCTION

Procedural sedation and analgesia (PSA) has been shown to be a safe and effective relief for pain, anxiety and discomfort during procedures performed in the emergency department (ED).^1^,^2^ Capnography is advocated to measure expired carbon dioxide and to assess ventilation adequacy.^3^ In 2014, the American College of Emergency Physicians (ACEP) recommended routine use of capnography during PSA and issued a Level B recommendation, citing studies demonstrating capnography effectiveness in early detection of hypoventilation.^4^ Despite widespread adoption in academic centers, the use of capnography in community hospitals has not been characterized. Quantifying the penetrance of this practice may lend insight into potential barriers to implementation of new technology advocated in national guidelines. We aimed to measure PSA capnography implementation in EDs within a Midwestern state, and to describe factors associated with PSA and continuous capnography adoption.

## METHODS

### Study Design and Population

This study was a cross-sectional telephone survey of ED nurse managers and designees in Iowa EDs from May 2017 to June 2017. We identified all Iowa facilities using the Iowa Hospital Association hospital database (n=121).^5^ ED nurse managers, designees, and hospital recruiters were acquired through telephone interviews. Information regarding ED volume and a hospitals designation by the Centers for Medicare and Medicaid Services (CMS) was extracted from Iowa Hospital Association databases. The study was determined not to be human subjects research by the institutional review board.

### Data Collection, Sources, and Definitions

The questionnaire and telephone prompts were designed by the study team, which included two board-certified emergency physicians (EP) with rural emergency medicine (EM) and health services research expertise. The questionnaire included hospital infrastructure, physician staffing, and critical intervention capabilities. We defined “capability/capable” as having the infrastructure, staffing and training necessary to perform a given intervention. The complete questionnaire can be found in the online supplemental Appendix. No validation study was conducted because elements of the survey instrument were objective facts.

We collected hospital-level variables from these Iowa Hospital Association datasets:*“Iowa Hospital Data”* and *“Services Directory”*.^5^ “Hospital classification” is determined by the CMS based on an institution’s bed volume, access to specialty services, and proximity to highway infrastructure and surrounding institutions. “Average ED volume” was calculated as the mean, self-reported annual ED census between 2013 and 2015, and were grouped into quartiles. Staffing models included whether EPs were board-certified in EM, family practice (FP), or both, and whether advanced practice providers (APPs, defined as physicians’ assistants and nurse practitioners without a physician physically present) were used.

### Outcome

The primary outcome was use of PSA capnography.

### Analysis

We characterized PSA capnography use descriptively. We then conducted univariate analyses to measure the association of capnography use with hospital factors (e.g., CMS designation and rurality, ED volume quartile, and staffing models). Proportions, relative risks, and 95% confidence interval [CI] are reported. All analyses were completed using SAS version 9.4 (SAS Institute, Cary, North Carolina).

## RESULTS

### Survey Results and Descriptive Analysis

A total of 118 hospitals of the 121 identified provided data (response rate = 98%), with staffing data acquired for 102 of these 118 hospitals (response rate = 86%). The majority of EDs in the state were rural (n= 93, 78%), with a median of 5,057 ED visits per year (interquartile range [IQR], 2,823–14,322). Median ED volumes ranged from 1,992 to 29,329 between the first and fourth quartiles, respectively. Approximately half of the hospitals had FP providers only, compared to 16% with board-certified EPs only (Table).

Nearly all hospitals (n=114, 97%) reported using continuous capnography for ventilated patients, and most (74%) reported use of capnography during PSA (n=87). Approximately 25% (n=29) reported using capnography exclusively for ventilated patients. Only two institutions reported use of PSA capnography without continuous capnography for ventilated patients. The distribution of staffing patterns and CMS designation class, stratified by PSA capnography use, is presented in the figure.

### Univariate Analysis

Urban hospitals were more likely to use PSA capnography than critical access hospitals (relative risk ratio 1.45, 95% CI, 1.22–1.73). Use of PSA capnography increased with ED volume, as hospitals in the highest quartile were 1.44 times more likely to use this compared to those in the lowest quartile. When compared to facilities with FP providers only, those with board-certified EPs only were 1.46 (95% CI, 1.15–1.87) times more likely to use PSA capnography. There was no difference in PSA capnography use between facilities with both providers and those with only FP physicians.

## DISCUSSION

At the hospital level, there are many factors that influence the decision-making process to adopt or reject a new professional policy. In a rural Midwestern state, where the majority of EDs are situated within small and financially vulnerable critical access hospitals, investment in new technology can take longer than it does in urban counterparts.^6^ Comparing this with implementation of other new technologies [e.g., electronic health records (EHR)], may provide a useful theoretical framework to understand disparities.^7^ For example, previous studies have demonstrated that the early adopters of EHR were typically large, private, urban and teaching hospitals, similar to our findings. In recent years rural facilities closed the gap in EHR implementation, but disparities remain when considering advanced measures of “meaningful use” of EHR, an advanced metric used by CMS to quantify the integration of EHR into healthcare delivery.^8^ Similarly, nearly all hospitals (97%) in Iowa use continuous capnography to monitor intubated patients, and most (74%) used capnography for procedural sedation.

Although there are significant differences between implementing a new EHR system and a new monitoring technique, the principles behind the decision-making process are the same. Kruse’s systematic review of EHR implementation included practical and theoretical considerations for or against adopting EHR systems.^7^ From this, applicable factors to PSA capnography implementation may include patient volume/complexity, financial considerations, professional support, provider comfort, hospital location, and regional interdependence.

While our study cannot elucidate the importance of these factors on a facility’s decision to implement capnography for PSA, our data demonstrate several supportive trends. Hospitals that exclusively hire board-certified emergency physicians, who may be more likely to have trained with these tools, were more likely to have access to capnography for PSA. Additionally, higher patient volume and urban centers were associated with increased capnography use. This may be due to management of higher acuity patients who may benefit the most from capnography monitoring. It is important to consider that there are likely collinear relationships between several of the variables measured; among facilities in the highest ED volume quartile, 93% had board-certified EP staff (48% board-certified EPs exclusively, 45% both board-certified EPs and FP physicians). As expected, Medicare class was highly correlated (r=0.74) with ED volumes.

## LIMITATIONS

While we were able to describe the availability of certain resources to an institution, this study was not designed to evaluate the frequency with which these resources were used and for what indications. For example, while sites may have indicated that physicians use capnography for PSA, there may have been variability with regard to the frequency of use (i.e., continuous vs intermittent), as well as variation in the physicians within the same facility. The focus of this study, however, was to identify availability of this procedure at a minimum. A cursory review of the existing literature yielded no substantive studies describing the prevalence of procedures requiring conscious sedation in rural and critical access hospitals. This study focuses on common characteristics of institutions across a state, and cannot be used to ascertain the rationale of an individual institution to adopt or not adopt a clinical practice.

## CONCLUSION

Continuous capnography for the monitoring of ventilated patients is found in nearly all EDs, independent of size, location, patient volume, or staffing practice. Capnography for the use of PSA, however, is less likely to be found in smaller, rural departments. Future research is needed to describe the services provided by rural and critical access institutions to better understand the extent to which ACEP professional policies may be feasibly implemented within a rural setting.

## Supplementary Information



## Figures and Tables

**Figure:**
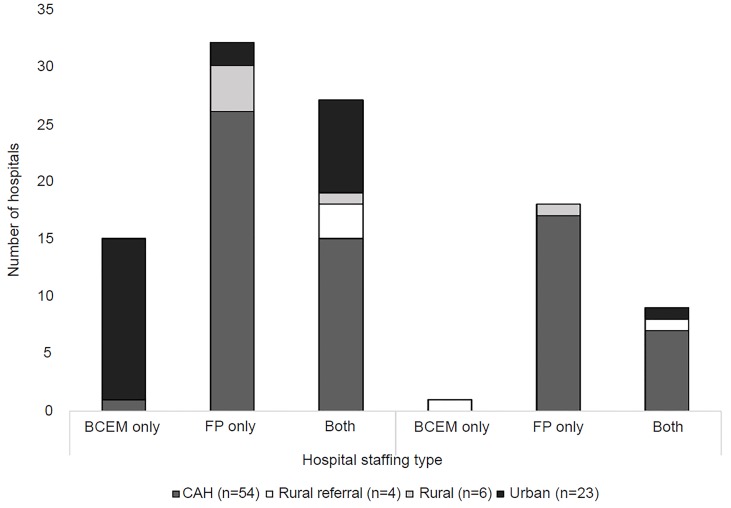
Distribution of PSA capnography use by staffing patterns and CMS classification scheme. *PSA*, procedural sedation and analgesia; *CMS*, Centers for Medicare and Medicaid Services; *BCEM*, board-certified emergency medicine; *FP*, family practice; *CAH*, critical access hospital.

**Table:** Summary of hospital characteristics.

Characteristics	Overall (n=118)		Procedural capnography available (n=87)		Procedural capnography unavailable (n=31)		Relative risk	
			
	N	%	N	%	N	%	RR	95% CI
Centers for Medicare and Medicaid services designation and rurality								
Critical access hospital	80	67.8	53	60.9	27	87.1	Ref	
Rural referral hospital	6	5.1	4	4.6	2	6.5	1.01	0.56–1.81
Rural hospital	7	5.9	6	6.9	1	3.2	1.29	0.92–1.82
Urban location/hospital	25	21.2	24	27.6	1	3.2	1.45	1.22–1.73
Staffing^1^								
FP only	50	49.0	32	36.8	18	58.1	Ref	
BCEM only	16	15.7	15	17.2	1	3.2	1.46	1.15–1.87
Both FP and BCEM	36	35.3	27	31.0	9	29.0	1.17	0.89–1.55
APP in solo coverage	37	36.3	24	27.6	13	41.9	0.84	0.64–1.11
ED volume								
Lowest quartile	29	24.6	18	20.7	11	35.5	Ref	
2nd quartile	30	25.4	21	24.1	9	29.0	1.13	0.78–1.63
3rd quartile	30	25.4	22	25.3	8	25.8	1.18	0.83–1.69
Highest quartile	29	24.6	26	29.9	3	9.7	1.44	1.06–1.97

1 Percents for staffing represent total within each category among hospitals with available data

*FP*, family practice; *BCEM*, board-certified emergency medicine; *APP*, advanced practice provider; *CI*, confidence interval; *ED*, emergency department; *RR*, relative risk.
