# Supplementary material for: End-tidal CO2 Monitoring is Available in Most Community Hospitals in a Rural State: A Health System Survey
Source: West J Emerg Med. 2019 Feb 14;20(2):232–6. doi: 10.5811/westjem.2018.12.40554 (PMC6404716; doi:10.5811/westjem.2018.12.40554)
Supplement: Supplementary file 1 [file wjem-20-232-s001.pdf]

Supplemental Appendix – Capabilities and Infrastructure Questionnaire

Hosp ID#:

Contact:

Research Questionnaire

		YES		NO	
The following questions are in regards to departmental assets available to your facility:					Mid
**1.**	Does your facility have a physician on-site 24 hours per day?	○		○	○
**2.**	Does your facility have an intensive care unit?	○		○	
**2a.**	***If yes***: How many beds?				
**3.**	Are there dedicated critical care physicians on staff?	○		○	
**4.**	Is there a critical care physician accessible 24 hours a day?	○		○	
**5.**	Does your facility have any of the following specialty services accessible 24 hours a day, only for outpatient consults or not at all?:	**24h**	**Otpt**	**No**	**Rural Outreach**
**5a.**	Cardiology	○	○	○	○
**5b.**	Nephrology	○	○	○	○
**5c.**	General Surgery	○	○	○	○
**5d.**	Infectious Diseases	○	○	○	○
**5e.**	Internal Medicine	○	○	○	○
**5f.**	Family Medicine	○	○	○	○
**5g.**	Gastroenterology	○	○	○	○
**6.**	Does your facility have access to Telemedicine services? ***If yes:*** which services?	○		○	
**6a.**	Critical Care	○		○	
**6b.**	Emergency Medicine	○		○	
**6c.**	Others: ___________	○		○	On-Call?
**7.**	Is there a pharmacist on-site 24 hours a day?	○		○	○
The following questions are in regards to your facilities ability to perform specific interventions:					Last Month?
**8.**	Is your facility capable of managing a patient on a mechanical ventilator for longer than 24 hours? ***If yes:*** *Performed in the last month?*	○		○	○
**9.**	Is your facility capable of administering vasopressor therapy for longer than 24 hours? ***If yes:*** *Performed in the last month?*	○		○	○
**10.**	Does your facility perform hemodialysis?	○		○	
**11.**	Is your facility capable of placing central lines? ***If yes****: Have they been placed in the last month? Who places?*	○		○	○
**11a.**	Intensivist	☐			
**11b.**	Emergency Physician	☐			Surgeon?
**11c.**	Generalist	☐			☐
**11d.**	Advanced Practice Provider	☐			
**12.**	Are antibiotics stocked in your Emergency Department?	○		○	
**13.**	Does your facility use continuous capnography while monitoring an intubated patient?	○		○	
**14.**	Is capnography used during procedural sedation in the Emergency Department?	○		○	
These next questions are in regards to your facilities institutional protocol and quality improvement:					
**15.**	Does your facility implement a specific sepsis protocol, care plan, or order set?	○		○	
**16.**	Does your facility participate in a sepsis-specific quality improvement initiative?	○		○	

**16a.**	***If yes***: Does your facility specifically track adherence with sepsis bundles or track sepsis outcomes?	○	○
**17.**	Does your facility implement an automated sepsis screening algorithm in the electronic medical record?	○	○
These questions are in regards to the transfer of patients out of your facility:			
**18.**	Are there standardized criteria written or unwritten in place for transferring patients with sepsis?	○	○
**19.**	Are there any specific arrangements for transferring patients with sepsis to a specific hospital for further care?	○	○
This final question is subjective:			
**20.**	Is there a single intervention you believe would improve preparedness for acutely ill sepsis patients in your hospital?		
